# Fast and precise detection of litchi fruits for yield estimation based on the improved YOLOv5 model

**DOI:** 10.3389/fpls.2022.965425

**Published:** 2022-08-09

**Authors:** Lele Wang, Yingjie Zhao, Zhangjun Xiong, Shizhou Wang, Yuanhong Li, Yubin Lan

**Affiliations:** ^1^College of Electronic Engineering, College of Artificial Intelligence, South China Agricultural University, Guangzhou, China; ^2^Guangdong Laboratory for Lingnan Modern Agriculture, Guangzhou, China; ^3^National Center for International Collaboration Research on Precision Agricultural Aviation Pesticides Spraying Technology, Guangzhou, China; ^4^School of Agricultural Engineering and Food Science, Shandong University of Technology, Zibo, China; ^5^Department of Biological and Agricultural Engineering, Texas A&M University, College Station, TX, United States

**Keywords:** object detection, YOLOv5, ShuffleNet v2, litchi, yield estimation

## Abstract

The fast and precise detection of dense litchi fruits and the determination of their maturity is of great practical significance for yield estimation in litchi orchards and robot harvesting. Factors such as complex growth environment, dense distribution, and random occlusion by leaves, branches, and other litchi fruits easily cause the predicted output based on computer vision deviate from the actual value. This study proposed a fast and precise litchi fruit detection method and application software based on an improved You Only Look Once version 5 (YOLOv5) model, which can be used for the detection and yield estimation of litchi in orchards. First, a dataset of litchi with different maturity levels was established. Second, the YOLOv5s model was chosen as a base version of the improved model. ShuffleNet v2 was used as the improved backbone network, and then the backbone network was fine-tuned to simplify the model structure. In the feature fusion stage, the CBAM module was introduced to further refine litchi’s effective feature information. Considering the characteristics of the small size of dense litchi fruits, the 1,280 × 1,280 was used as the improved model input size while we optimized the network structure. To evaluate the performance of the proposed method, we performed ablation experiments and compared it with other models on the test set. The results showed that the improved model’s mean average precision (mAP) presented a 3.5% improvement and 62.77% compression in model size compared with the original model. The improved model size is 5.1 MB, and the frame per second (FPS) is 78.13 frames/s at a confidence of 0.5. The model performs well in precision and robustness in different scenarios. In addition, we developed an Android application for litchi counting and yield estimation based on the improved model. It is known from the experiment that the correlation coefficient *R*^2^ between the application test and the actual results was 0.9879. In summary, our improved method achieves high precision, lightweight, and fast detection performance at large scales. The method can provide technical means for portable yield estimation and visual recognition of litchi harvesting robots.

## Introduction

Litchi (Litchi chinensis Sonn.) is a subtropical evergreen fruit tree whose cultivation range has widespread worldwide. Its fruit is nutritious and tasty. Meanwhile, litchi is an important economic crop and has a high economic value. Today, China is one of the primary litchi producers, accounting for about one-third of the world’s annual litchi production. Litchi trees in China are mainly distributed in the southern hilly regions, and the annual output value of litchi-related industries is more than four billion US dollars (Qi et al., 2019).

The construction and implementation of smart orchards have become a current research focus. The detection and evaluation of fruit maturity are crucial for yield estimation and harvest in smart orchards. Currently, there are destructive and non-destructive methods for judging fruit maturity. Destructive methods are used to find the physicochemical or biochemical properties of fruits. They need high technical requirements and have to destroy materials. The detection speed is slow, but more phenotypic information can be obtained. In contrast, non-destructive methods have the advantages of lower cost, more reliable detection results, and no need to destroy the fruit (Arunkumar et al., 2021). Various non-destructive detecting methods for fruit maturity have been extensively studied, such as ultrasonic methods (Yildiz et al., 2018), near-infrared spectroscopy (Pissard et al., 2021), scanning laser Doppler vibrometers (Hosoya et al., 2017), magnetic resonance imaging (Srivastava et al., 2018), electronic nose (Calvini and Pigani, 2022), and so on. Although these methods have high detection accuracy, they are only suitable for a single fruit or laboratory detection. The scope and prospect of promotion among fruit farmers are small, and they are not suitable for judging large-scale fruit maturity and yield estimation in orchards. In addition, computer vision is also a non-destructive method that can be used for inspection and has been found to be applicable in precision agriculture. Fruits are accurately detected with the help of computer vision technology, after which the fruit harvest work is achieved using robot technology. This harvesting method is significant to the intelligent and automatic management of orchards (Tang et al., 2020; Wang and He, 2021; Qi et al., 2022). In present-day China, most regions rely mainly on manual litchi harvesting. Due to the strong seasonality of litchi ripening, it takes massive labor to complete the large-scale litchi fruit harvesting quickly. At the same time, the maturity of litchi during the growing period is inconsistent, and even litchi from the same orchard or the same tree are ripened in batches (Jin, 2020; Chen et al., 2022). Therefore, it is important to detect litchi and classify their maturity through computer vision technology, achieve litchi orchards yield estimation, and then guide harvesting robots or fruit farmers to unfold the picking efforts in a timely, selective, and batch manner. Among them, litchi detection is a prerequisite for realizing orchard yield estimation. It is an urgent issue to be solved in today’s orchard production.

Over the years, related scholars have discussed some traditional object detection methods for fruits, and the feature extractors used are often based on features determined by artificial prior knowledge, and it is difficult to achieve robust feature representation (Wang C. et al., 2022). Wan et al. (2018) used a back-propagation neural network to detect the maturity of fresh tomatoes. Dameshwari and Ravindra (2017) employed MATLAB software for defect identification and maturity detection of mango fruits. Khojastehnazhand et al. (2019) realized maturity detection and volume estimation of apricot with the help of image processing technology. Wang et al. (2017) studied four effective color components and six visual features commonly used in image recognition, trained the Bayesian, KNN, ANN, and SVM classifiers, and finally integrated them to realize litchi recognition. However, the disadvantage of this method is that the detection algorithm requires a long reasoning time. He et al. (2017) used an improved LDA classifier to detect green litchi per plant. However, the parameters used in the multi-stage processing need to be manually specified, and the parameter debugging process is too cumbersome to be widely promoted. Xiong et al. (2018) proposed a nighttime litchi identification method based on Otsu, but the method requires a single environment and is not suitable for litchi recognition in a natural environment.

In recent years, there have been some two-stage fruit detection methods with the development of deep learning. Among them, the Faster R-CNN is widely applied as a classical algorithm (Barnea et al., 2016; Sa et al., 2016; Fu et al., 2020). Apolo-Apolo et al. (2020) employed the Faster R-CNN to detect citrus images captured by Unmanned Aerial Vehicles and then estimated citrus yield with an average standard error of 6.59%. Gao et al. (2020) achieved multi-class fruit detection with the help of the Faster R-CNN, such as apples. Fu et al. (2018) implemented a two-stage detection of kiwifruit fruit images using Faster R-CNN and ZFNet network. The average accuracy of the model was 92%, and the average processing time for a single image was 0.27s. Vasconez et al. (2020) applied the Faster R-CNN with the Inception V2 network implementation to detect avocado, lemon, and apple under different field conditions, with an mAP of 93%, and it took 0.22s on average to process a single image. Although the two-stage object detection method shows good performance, its detection speed has limitations that make it difficult to be applied to field real-time detection.

To meet the requirements of real-time object detection under complex agricultural application scenarios, it is usually necessary to seek an optimum between detection accuracy and calculation time. The single-stage object detection algorithm represented by the YOLO series has achieved a better balance between accuracy and speed. Koirala et al. (2019) improved the YOLOv3 network for mango detection by merging feature maps of different resolutions from the middle layer. The mAP of 98% was achieved, and the model took 0.07 s for a single image. Liu et al. (2020) replaced the traditional rectangular bounding box of the YOLOv3 model with a circular bounding box for tomato detection, achieving an mAP of 96% and a detection speed of 0.054 s on an image of 3,648 × 2,056 pixels. Liang et al. (2020) adopted the YOLOv3 model for detecting litchi fruits in a natural environment at night while extracting a region of interest on the main stem of litchi. Tian et al. (2019) combined YOLOv3 and DenseNet to detect apples in orchards with an F1 score of 0.817, IoU of 0.896, and a processing time of 0.304 s for a 4,000 × 3,000 pixel image. Wu et al. (2021) put forward an improved YOLOv3 model based on clustering optimization for multi-object recognition of banana buds and inflorescence axes. Zheng et al. (2021) improved the framework of YOLOv4 and proposed a multi-scale convolutional neural network based on a bidirectional feature pyramid network to detect green citrus with an accuracy rate of 91.55%. Wang L. et al. (2022) trained the improved YOLOv4 model to detect dense plums. Compared with some results from the original YOLOv4 model, the model size of the improved model was compressed by 77.85%. The parameters were only 17.92% of the original model parameters, and the detection speed were accelerated by 112%. Li D. et al. (2021) proposed to use a MobileNet-YOLOv4 model for fast and accurate detection of longan strings in Unmanned Aerial Vehicles images. Nevertheless, further studies are needed for highly occluded and overlapped fruit objects.

Litchi grows in haphazard and seriously adhered clusters in the natural environment, which branches and leaves may block. In litchi images used for yield estimation, minor pixel points on litchi fruits can make insufficient extraction of important feature information, resulting in missed or false detection. Although it is more convenient to carry out long-range detection with Unmanned Aerial Vehicles, only litchi distributed in the superficial layers are detected, and fruits that are obscured or overlap may be missed, which would give low yield estimates (Peng et al., 2022). Object detection methods based on deep learning have shown good performance on public datasets, but these models do not consider processing small, dense objects. When facing small object litchi that is densely obscured, problems such as insufficient feature extraction often occur. While with larger-scale detection feature maps, detection speed becomes a bottleneck of the model. In addition, although some lightweight fruit algorithms based on edge devices have also been studied (Zhang et al., 2021), deploying deep learning algorithms in the real-time field remains challenging. In this case, according to the biological characteristics of litchi fruit, our object detection algorithm needs to take into account the detection speed while considering the problem of densely occluded small objects. Consequently, how to improve the accuracy and speed of litchi object detection under dense and high overlaps becomes an essential goal of this study.

Since You Only Look Once version 5 (YOLOv5) model has been proven to perform well in other application domains to detect small objects (Chen et al., 2021; Zhang et al., 2022). In response to the above problems, this study will carry out improvement work based on the YOLOv5 model regarding the accuracy, model size, and detection speed to obtain a deep learning model suitable for litchi fruit detection. The specific improvements include: (1) increasing the recognition capability of the model for small objects by changing the input size of the model; (2) enhancing the accuracy of model detection by replacing and fine-tuning the backbone feature extraction network and adding an attention mechanism; and (3) further reducing the model parameters and accelerating the reasoning time of the model by deleting the detection head. Finally, an Android application based on the improved model will be developed to obtain the maturity and yield information of litchi conveniently and quickly by mobile phone. In conclusion, the improved method presented in this paper achieves high performance and fast detection performance in litchi orchard environments, providing technical means for portable litchi yield estimation and visual recognition of litchi harvesting.

## Materials and methods

### Materials

#### Image data acquisition

**FIGURE 1:**
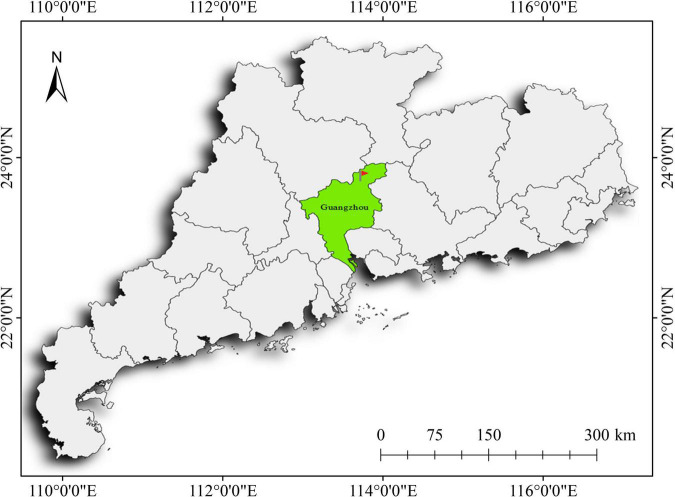
The geographical location of the image acquisition.

RGB images of litchi used in this study were all taken from the Litchi Exposition Park in Conghua District, Guangzhou City, Guangdong Province, China (2334′60″*N*, 11337′12″E), and the location is shown in Figure 1. All images were acquired between 9:00 a.m. and 6:00 p.m. on May 17, June 11, and June 30, 2021. The acquisition device used is a smartphone with three postfixed cameras: a 40-megapixel main camera, a 12-megapixel ultra-wide-angle camera, and an 8-megapixel telephoto camera. The 40-megapixel main camera at a pixel density of 400 ppi was chosen for this study. The sensor model is Sony IMX600 with CMOS photosensitive chip. The lens uses an f/1.6 aperture and an RYYB filter. The device supports optical image stabilization and the autofocus in three modes (laser focus/phase focus/contrast focus). In this study, the image’s resolution was set to 3,648 × 2,736 pixels, and the exposure parameter was set to auto mode. Finally, the images were saved in JPEG format.

In the natural growth state, litchi grows in dense clusters for litchi orchards. Immature litchi is turquoise and close to the branches and leaves. Mature litchi appears red. To capture as many images of litchi fruits in the natural environment as possible under multiple weather conditions, the acquisition equipment randomly transformed the sampling angle within a 2–5 m imaging distance. A total of 1,375 original litchi images were collected in this study, and litchi fruit samples with different maturity, posture, size, background, density, and occlusion were included in this dataset. Table 1 shows the sample collection site’s weather conditions and quantity distribution during the collection period.

**TABLE 1:** The weather conditions and quantity distribution during the image collection period.

Date	Weather conditions	Number
May 17, 2021	Rainstorm to cloudy	375
June 11, 2021	Sunny to cloudy	500
June 30, 2021	Sunny and breezy	500

#### Building the dataset

**FIGURE 2:**
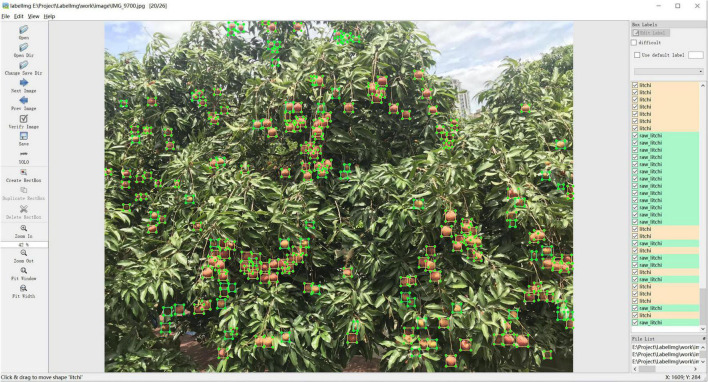
Data annotation example: the blue box represents mature litchi, and the yellow box represents immature litchi.

At the data processing stage, litchi fruits were divided into two classes according to their maturity: mature litchi (litchi) and immature litchi (raw_litchi). As shown in Figure 2, LabelImg software was used to manually annotate the ground location boxes and class of litchi fruits in the original image and generated the corresponding annotation files. After the image annotation work was completed, the entire dataset was randomly divided into a training set, validation set, and test set according to the ratio of 7:1:2 for subsequent training and testing of the model. Statistically, each image collected in this study contains 10–100 fruits, and there are 53,855 labels for the litchi fruits dataset. The class ratio between the two is about 1.28, illustrating no significant data imbalance within the dataset.

Before the training of the model, this study performed several random combinations of offline data augmentation methods such as flipping, clipping, rotation, scaling, translation, brightness, histogram averaging, salt and pepper noise, and Gaussian noise on the training set (Taylor and Nitschke, 2018; Lyu et al., 2022). The five data augmentation methods were specifically (1) random horizontal flip of 25% of the training set + random vertical flip of 25% of the training set + random crop of 0–20% region of the image width/height; (2) histogram averaging + pepper noise 2%; (3) rotation 10°; (4) random modification of the brightness to 50–150% of the original + random Gaussian noise; (5) random scaling transformation 70–95% + random panning (−15–15%). To guarantee the quality of the data annotations before and after data augmentation, the anchor box positions in the annotation files associated with the original images were also coordinately transformed with different data augmentation methods. As shown in Table 2, the number of training sets after data augmentation was enlarged by five times, yielding a total of 5,710 image data available for network training. Ultimately, there are 6,175 image data for the litchi dataset.

**TABLE 2:** Details of the litchi image dataset.

Dataset	Number of original images	Number of augmented images
Training set	962	5772
Validation set	138	138
Test set	275	275
Total	1375	6175

### Methodologies

#### YOLOv5 model

YOLOv5 (Glenn et al., 2021) is one of the YOLO series of networks and an improved version of YOLOv3 (Redmon and Farhadi, 2018). The idea of the YOLO series of networks is to convert the object detection problem into a regression problem. Using the CNN network to process the image can directly obtain the class and position coordinates of the object, which makes the model have high-performance results in detection accuracy and speed. Compared to the YOLOv4 model (Bochkovskiy et al., 2020), the YOLOv5 model has achieved a better balance between accuracy and speed. The YOLOv5 model consists of multiple versions of different scales, from which extended lightweight model versions can be deployed on various devices.

The network structure of YOLOv5 is composed of a backbone network, a neck network, and a detection head. The YOLOv5 model employs the new CSP-Darknet53 structure as the backbone network to extract image features. The structure contains CBS modules, the C3 modules, and the SPPF module. CBS indicates the synthesis module of Conv, BN, and SiLU activation functions. The C3 module is the main module for residual feature learning, and its structure is divided into two branches. One is stacked by multiple Bottlenecks and three standard convolution layers, and the other only passes through a basic convolution module. Finally, the two branches are merged. The SPPF module further integrates multiple parallel MaxPool2d of different sizes, which can solve the multi-scale object problem to a certain extent. When the size of the input image is 640 × 640, the feature maps of 80 × 80 × 128, 40 × 40 × 256, and 20 × 20 × 512 are output after passing through the backbone network. As the neck of the network, the path aggregation network (Liu et al., 2018) plays the role of feature fusion and aggregates information paths in a combined bottom-up and top-down manner to obtain richer features from each layer. Like the YOLOv3 model, the YOLOv5 model also adopts three scales of head to detect small (80 × 80 × 128), medium (40 × 40 × 256), and large objects (20 × 20 × 512), respectively. Finally, these feature maps are divided into grids, and the K-means algorithm is used for each grid to generate anchor coordinate boxes to predict object boundaries iteratively. Each detection box outputs a feature vector of predicted bounding box center coordinates(*x*, *y*), width, height, confidence score, and class probability. To prevent a single object from generating redundant or overlapping prediction boxes, a Non-Maximum Suppression threshold is set to determine the final detection box. The last detection result of the input image is rescaled to the original image size, enabling the detection of the object.

#### Lightweight backbone network

Typically, the deep learning model has a large number of parameters and computations, requires high computer requirements, and is challenging to run directly on mobile phones or other edge devices. In this context, Ma et al. (2018) proposed a computationally efficient and lightweight CNN model suitable for mobile devices—ShuffleNet v2. The research results show that ShuffleNet v2 has higher accuracy than MobileNet v2 (Sandler et al., 2018) and Xception (Chollet, 2017) under the same model complexity. The contribution of ShuffleNet v2 lies in that it summarizes four rules of network design according to the performance of the actual scenarios, namely: (1) keep the number of input and output channels of the convolution layer equal to minimize memory access cost; (2) reduce group convolution operations to reduce memory access cost; (3) reduce network branching structures to enhance parallel computing power; and (4) reduce element operations to speed up the network speed.

**FIGURE 3:**
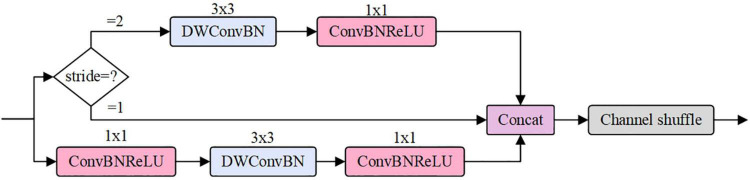
ShuBlock module.

The ShuBlock module is the basic unit of ShuffleNet v2 (Figure 3). The module has two branches. A branch is a residual unit containing a 1 × 1 convolution, a 3 × 3 deep separable convolution, and a 1 × 1 convolution. The other one needs to be handled in two cases: if the stride is 1, the residual edge is a shorted connection branch; if the stride is 2, the residual edge is a branch composed of a 3 × 3 deep separable convolution and a 1 × 1 convolution. Finally, the two components are stacked together, and a Channel shuffle is introduced to achieve information exchange between channels.

#### Convolution block attention module

The Convolution Block Attention Module (CBAM) is an attention mechanism that combines the Channel Attention Module (CAM) with the Spatial Attention Module (SAM) (Woo et al., 2018). It is often seamlessly integrated into some CNN networks for end-to-end training due to its lightweight and generalized characteristics. Its function is to strengthen the model’s ability to extract features and suppress invalid background information by refining the input feature map (Li X. et al., 2021).

**FIGURE 4:**
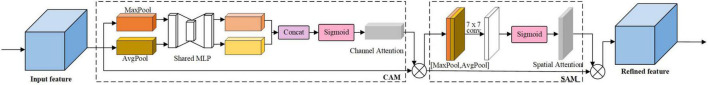
Convolution block attention module structure.

As shown in Figure 4, the CBAM structure is a tandem of two sub-modules of the channel and spatial attention, and the input of SAM is the feature map modified by the CAM mechanism, which can obtain more comprehensive and reliable attention information. The feature matrix is first input to the channel attention sub-module, where each channel represents a feature detector. The role of the CAM here is to process feature maps from different channels and focus on the meaningful feature map information. After that, the channel compression weight matrix is output, then multiplied by the original input feature characteristic matrix. When the feature map adjusted by CAM enters the spatial attention sub-module, the SAM will process the feature region of meaningful information in the feature map, generate the spatial compression weight matrix, and perform the same multiplication operation. And finally, the refined feature map is obtained. The CAM and SAM modules selectively fuse deep and shallow features. High dimensional features guide low dimensional features for adaptive feature refinement of CAM, and low dimensional features guide high dimensional features in reverse for the screening of Sam. This sequential way improves the network model’s ability to extract features without significantly increasing the amount of computation and parameters.

In Figure 4, MaxPool represents the maximum global pooling; AvgPool represents global average pooling; Share MLP represents a multi-layer perceptron with shared weights; Channel Attention indicates the channel attention map output by CAM; Conv indicates convolution operation; Spatial Attention denotes the spatial attention map.

### The improved YOLOv5s network structure

The YOLOv5 model is classified into x, l, m, s, and n versions according to the complexity of the network structure. To better balance accuracy and speed, this study chose the 6.0 version structure of YOLOv5s as the basis for model improvement. This study will investigate the accuracy, model size, and detection speed to find a more suitable litchi fruit detection model. Specifically, we will first replace and fine-tune the backbone network structure to compress the model size. Then we will add an attention mechanism to strengthen the accuracy of the model detection. After that, we will change the input size of the model from 640 × 640 pixels of the original YOLOv5 model to 1,280 × 1,280 pixels to improve the model’s ability to recognize small objects. And finally, we will reduce the model parameters and computation to accelerate the detection speed of the model by removing the large-scale detection head.

#### Fine-tuning the lightweight backbone network

This study used the building blocks of ShuffleNet v2 to form the backbone network of the improved model. First, the 6 × 6 convolution method from the first layer of the original backbone network was retained, and the first layer convolution of the ShuffleNet v2 was also changed from 3 × 3 to 6 × 6. Second, the successive stacks of ShuBlock building blocks are used to construct the lightweight backbone network. In this way, the number of model parameters can be reduced as much as possible to ensure the feature extraction capability. Afterward, like the original model, the SPPF structure was used on the output of the last layer to strengthen the extracted features. Therefore, when the size of the input image is 1280 × 1280, the output feature maps of (160 × 160 × 116), (80 × 80 × 232), and (40 × 40 × 464) will be obtained after the improved backbone network.

#### Optimize the network structure

In the feature fusion stage, low dimensional feature maps were introduced to increase the feature information of small objects while raising a large amount of background noise, impairing the accuracy of hierarchical object detection. To this end, we added a CBAM module to gain adequate feature information and suppress background noise before the feature maps entered the neck network.

**FIGURE 5:**
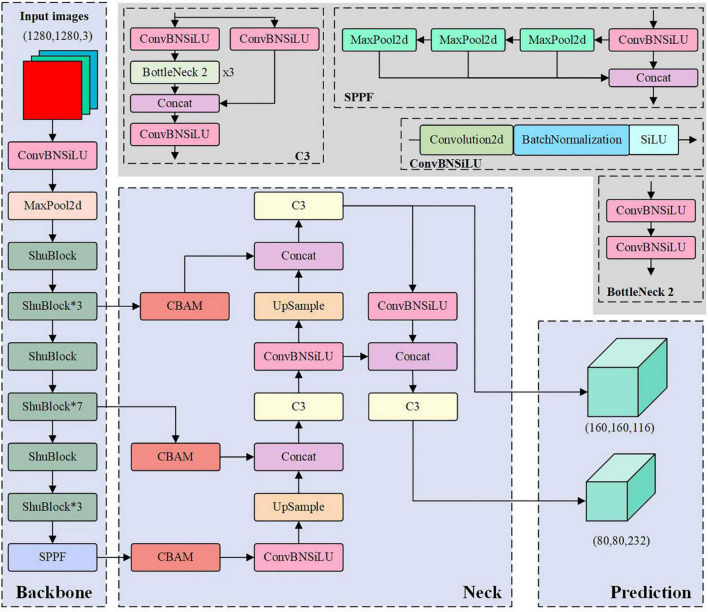
Network structure diagram of the improved model.

For the three detection heads output by the original YOLOv5 model, the size(20, 20, 512) is used to detect large-scale objects. For the small object litchi in this study, the large-scale detection head had little contribution to the recognition results. Therefore, to further simplify the complexity of the detection model, this paper removed the 21st–23rd layers of the original YOLOv5 network and the detection output head (20, 20, 512) mimicking the network structure of the YOLOv4-tiny model (Wang et al., 2021). The pruned model has only two detection heads. This pruning operation gave the shallow feature map a smaller receptive field, which is more suitable for identifying dense small object litchi. Figure 5 shows the network structure of the improved model and the illustrations of each specific module, which are distinguished by different colors. In Figure 5, the blue area indicates the network structure of the improved model, and the gray area shows the illustration of each specific module that appears in the improved model. Where Conv is convolution; Concat is a feature fusion method based on the addition of channel numbers; BN is Batch Normalization; UpSample is upsampling; BottleNeck indicates bottleneck layer. The size of the input image is a tensor with dimensions of 1,280 × 1,280 × 3, and the final convolution operation will form image tensors with dimensions of 80 × 80 × 232 and 160 × 160 × 116.

### Training of litchi object detection model

To make the model learn more valuable features, the input image of the model was first adjusted to 1,280 × 1,280 pixels, and the image padding method was applied to maintain the aspect ratio of the original image. After that, we improved the model according to the proposed improvement method. During this process, the loss function of the original YOLOv5s model was not altered. Finally, the annotated litchi training set was trained to utilize the Pytorch deep learning framework, and the validation set was used to verify the effect and performance of the model training.

The experimental environment in this study is shown in Table 3. First, the annotated VOC format dataset was converted to the data format accepted by the YOLOv5 model. Second, the parameters of the model training process need to be configured. After that, it is time to turn on the improved object detection network training. The training parameters of the improved model are: the initial learning rate is set to 0.01, the eta_min is 2 × 10^−3^, the last_epoch is −1, the momentum parameter is 0.937, the delay parameter is 5 × 10^−3^, the batch size is set to 8, and the T_max is 250. Optimized by the AdamW optimizer during the training process. Eight workers were employed for multi-threaded model training, and the cosine annealing learning rate was performed to update optimally during the training process. Besides offline augmentation methods, Mosaic data augmentation was also used to further enrich the background of detected objects, reinforce the awareness of litchi fruits characteristics, and strengthen the robustness and generalization performance of the model. The data-augmented network took about 28 h of training duration.

**TABLE 3:** The experimental environment in this study.

Name	Value
CPU	AMD R5-5600X 6-Core
Memory	32GB
Storage SSD	512GB
Graphics card	Nvidia RTX 2060 SUPER
Graphics memory	8GB
Operating System	Windows10
CUDA version	10.2
PyTorch version	1.7.1

## Experimental results and comparative analysis

### Model evaluation indicators

To precise evaluate the detection performance of the model on dense litchi images, we adopted eight evaluation indicators that are commonly used in classical object detection algorithms: Precision (P), Recall (R), F1-score, average precision (AP), mean average precision (mAP), network parameters, model size, and detection speed. And the IoU value was 0.5 during the experiment. This study used frames per second (FPS) to evaluate the model’s real-time detection performance. The larger the FPS, the faster the model detection speed. Each formula of P, R, F1, AP, and mAP is shown in Equations (1–5).


(1) $$P=\frac{TP}{TP+FP}$$

(2) $$R=\frac{TP}{TP+FN}$$

(3) $$F1-score=\frac{2PR}{P+R}$$

(4) $$AP\left(k\right)=\int_{0}^{1}P\left(R\right)dR$$

(5) $$mAP=\frac{\sum_{1}^{Q}AP\left(k\right)}{Q}$$

In the above formulas, *Q* is the total number of classes; *AP*(*k*) denotes the *AP* value of the *k*_*th*_ class, *k* = 2 in this study; TP represents the number of litchi fruits correctly detected (true positive); FP indicates the number of false detected (false positive); FN represents the number of missed detection (false negative). F1-score is defined as the harmonic mean of model precision and recall. *AP* represents the area of a PR curve and coordinate axis drawn according to different thresholds. It is the standard to measure the model’s sensitivity to an object. The higher the *AP* value, the better the performance of the object detection algorithm. The mAP is the average value of multiple *AP* classes, representing the universal detection performance of the algorithm for all classes. Compared with the F1-score, mAP is an indicator that more closely reflects the global performance of the network.

### Experimental results and analysis

#### Experimental results

**FIGURE 6:**
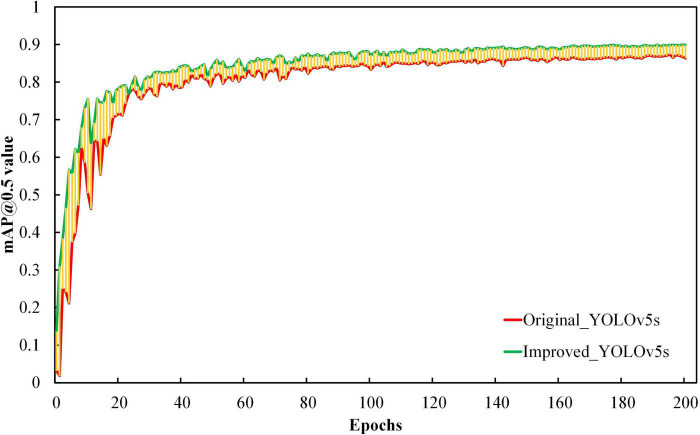
The comparison of mAP@0.5 of the model before and after improvement on the training set.

As shown in Figure 6, we used the mAP@0.5 indicator to measure the model’s overall performance on the training set. The upward climbing speed of the curve changed from fast to slow before the 170th epoch. After the 170th epoch, the curve flattened gradually, and the slope slowly tended to 0. The result value of mAP@0.5 exceeded 90% during the period. In addition, comparing the change of the mAP@0.5 value of the model before and after improvement on the training set, the improved model consistently exceeded the mAP@0.5 value of the original YOLOv5 model, and the fluctuation between them is shown in the yellow shaded part in Figure 6. The above results illustrate that the improved YOLOv5s model performs better for litchi fruit detection.

Table 4 presents the evaluation results of the improved model on the test set consisting of 275 images. The experimental data shows that the improved YOLOv5s model performed on the test set with an overall mAP of 92.4%, F1-score of 0.87, model size of 5.1 MB, and average detection time of 78.13 frames/s (FPS ≥ 24), which meet the requirement of real-time detection. The gap between the precision and recall of each class ranges from −0.3 to 4.4%, and the AP values of mature and immature litchi are similar. In conclusion, the litchi detection model proposed in this study has the advantages of high precision, lightweight, and fast inference speed.

**TABLE 4:** Evaluation results of the improved model on the test set.

Class	P/%	R/%	mAP@ 0.5/%	F1- score	Model size/MB	FPS
litchi	88.1	88.4	93.9			
raw_litchi	87.1	82.7	90.8	0.87	5.1	78.13
all	87.6	85.6	92.4			

#### Detection effect of the improved model in different scenarios

Precisely detecting the obscured litchi fruits before harvesting is significant for litchi yield estimation. Most litchi tree grows in open and unstructured mountain orchards. Factors such as uncertain light exposure, occlusion, and clustered aggregation of fruits, especially the color similarity between immature litchi fruits and the background, make detecting all fruits on litchi trees in a natural environment very challenging.

**FIGURE 7:**
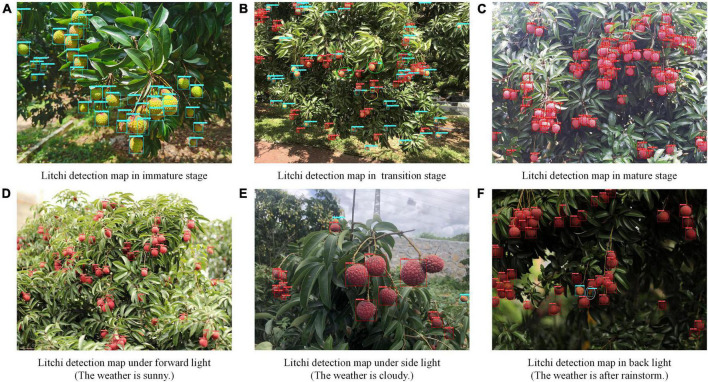
The improved model’s detection results at different maturity stages. Where the white oval indicates that the fruit was falsely detected; the yellow oval represents the missed detected fruits; the green oval represents that the model is temporarily unable to distinguish whether it is immature or mature litchi.

To investigate the influence of different maturity stages and light conditions on the litchi fruit detection model, we show litchi’s image detection results in Figure 7. Where the white oval indicates that the fruit was falsely detected; the yellow oval represents the missed detected fruits; the green oval represents that the model is temporarily unable to distinguish whether it is immature or mature litchi.

As presented in Figure 7, the improved model has a high recognition accuracy for litchi fruits in the mature (Figure 7A) or immature stage (Figure 7C). For the transition period of litchi fruits, the improved model shows good recognition performance, but there is one missed detection (as marked by the yellow oval in Figure 7B) and three places where the maturity could not be determined (as denoted by the green oval in Figure 7B). After alignment of detected results, it is found that the accuracy values judged by the model as mature litchi are all higher than those of immature litchi. Because the color distinction of the semi-mature fruits in the middle or later stages of veraison is not significant, the classification results of the model are rocked between mature and immature. This situation will be solved by further refining litchi categories in future research.

The model can clearly recognize fruits in the forward and side light conditions with high identification values (Figures 7D,E). The fruit characteristics are clear and stable in the forward and side light conditions. The contour of the fruit area is well separated from the background so that even an indistinct fruit with wind jitter or camera jitter can be identified. The model exhibits a friendly performance for the identification of these two situations. Although fruits in the backlight condition are mostly recognizable, there appeared instances where the model falsely detected curled litchi leaves as immature fruits (as indicated by the white oval mark in Figure 7F). Without external interference, the model may only notice the approximate candidate box of the fruit without wrapping the entire fruit outline. Therefore, extracting fruit contour edge features under the backlight condition in natural orchards is highly challenging. Although the method proposed in this paper has some errors, it still accurately and sensitively completes the detection of the litchi fruit in the image.

**FIGURE 8:**
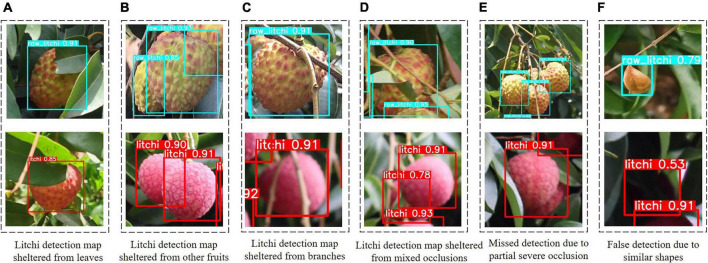
The detection effect of the improved model for various occlusion methods appearing in the dataset. **(A–D)** Indicates that the occluded litchi can be detected accurately by the improved model, and **(E–F)** indicates that the occluded litchi are not detected correctly by the improved model (false detection or missed detection).

To explore the detection performance of the improved model for occluded litchi fruits, we collected the detection clipping images that were occluded in various ways. Figures 8A–C are the clipping images only obscured by leaves, other litchi fruits, and branches. It can be seen from Figures 8A–C that the improved model has better recognition results for litchi fruits with single occlusion. Fruits mixed with multiple occlusion methods can also be identified by the improved model (Figure 8D), but multiple fruits with high overlap or severe occlusion can be mistaken for one fruit or directly missed (Figure 8E). The reason may be that litchi fruits grow tightly in clusters, some fruits are heavily occluded, and essential features such as contours are lost, making the predicted yield lower than the actual yield. There is another possibility that the collected litchi dataset is limited, and it is difficult for the model to use a limited dataset to traverse various occlusion cases in natural orchards. This problem will be solved by expanding the dataset in the future. As shown in Figure 8F, some shaded parts or curled dry leaves similar to the shape of the fruit may also be falsely detected as litchi fruits. The morphology of litchi trees in the natural environment is random and unstructured. The obstacles in front of the fruit are dense and three-dimensional, and the degree of occlusion from different perspectives is also different, causing the illusion of the existence of the fake fruit. The case will make the predicted output value higher than the actual output value. To solve this problem, the planting structure of plants needs to be changed in the future by increasing the tightness of the combination of agricultural machinery and agronomy so that the morphology of the litchi tree is more suitable for mechanized harvesting in agriculture.

**FIGURE 9:**
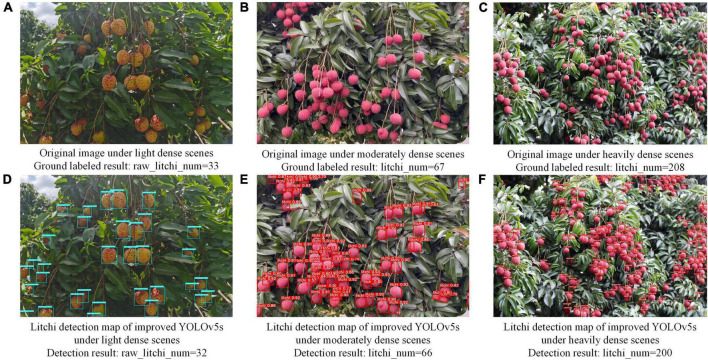
The detection effect of the improved model on litchi images with three density levels.

To evaluate the detection accuracy of the improved model for images with different densities, we randomly selected some images for experimental comparison research. Among them, for images below 40 litchi fruits, we considered it to be light dense. For images containing 40–80 litchi fruits, it is called moderately dense. For images where the number of litchi fruits exceeds 80, it is called heavily dense. The model detection results under three density levels are shown in Figure 9.

Figures 9A–C show the original images of litchi with three density levels, and Figures 9D–F are the corresponding litchi detection results. Longitudinally, only one missed detection occurred in the light and moderately dense images, and eight missed detections occurred in the heavily dense images. The missed detection rate was within 3%. After viewing the enlarged Figure 9F, it is known that two fruits missed detection because of the complete overlap of different sizes. There are three missed detections because of severe occlusion of leaves and incomplete splicing of fruit contour edges. The remaining missed detections resulted from distortions or blurring of the fruit caused by a long distance so that the improved model could not confirm the contour area of the fruit. The next step is sharpening the fruit’s contour using a motion blur super-resolution algorithm. In terms of horizontal comparison, the improved model shows better recognition performance on litchi images with different density levels and maintains a high number of recognitions and recognition rates.

### Ablation experiments

Based on the YOLOv5s model, we analyzed the influence of different improvement schemes on the detection performance of the model employing ablation experiments. All improvement operations were trained and validated using the same training and validation sets, and the tests were done on the same test set. The experimental results are shown in Table 5.

**TABLE 5:** The effect of different improvement schemes on the model performance.

Models	Image size	mAP@0.5 (%)	Parameters	Model size (MB)	FPS
YOLOv5s	640 × 640	88.9	7,015,519	13.7	104.17
+ ShuffleNet v2	640 × 640	87.1	3,680,751	7.42	119.05
+ CBAM	640 × 640	89.7	7,058,821	13.8	108.70
+ 1280	640 × 640	93.2	7,015,519	14.1	48.31
+ ShuffleNet v2 + CBAM	640 × 640	88.4	3,716,133	7.5	114.94
+ 1280 + ShuffleNet v2 + CBAM	1,280 × 1,280	92.5	3,716,133	7.95	56.82
+ 1280 + ShuffleNet v2 + CBAM + cut **(ours)**	1,280 × 1,280	92.4	2,251,496	5.1	78.13

Bold values indicates the final version of model improvements in this paper.

As shown in Table 5, the mAP of the original YOLOv5s model on 275 test images is 88.9%, the F1-score is 0.85, the number of parameters is 7,015,519, the model size is 13.7 MB, and the FPS is 104.17 frames/s. In contrast to the original model, the three improvement points proposed in this study positively impact different aspects. After replacing the backbone network alone, the model’s overall performance was slightly fine-tuned. It is worth noting that the model’s number of parameters and size is reduced to approximately half of the original model. When the CBAM module was added again, the mAP of the model showed a hint of elevation and a slight decrease in FPS values. After setting the input size of the model to 1,280 × 1,280 on this basis, the model gained more refined feature information. Its mAP reached 92.5%. The model size increased slightly with the enlarged image size, and the detection performance was significantly improved. After optimizing the network, the number of parameters continued to decrease when the overall mAP was similar, and the detection speed of the model accelerated by 37.5%. Compared with the original YOLOv5 model results, the mAP of the improved model presented a 3.5% improvement and 62.77% compression in model size. In summary, the improved method proposed in this paper achieves high-precision, small-scale, and fast detection performance at a large scale, which meets the requirements of real-time detection.

### Comparison with other deep learning models

Several more classical network models were selected for retraining in this study to investigate the performance differences between the improved model and other models. We adopted the control variables method to guarantee the reliability of the results. All models were trained on the same training set using the same training environment, and finally, the detection results of the network models were contrasted on the same test set.

The comparison results are shown in Table 6. The differences between the models are mainly reflected in mAP detection performance, model size, and detection speed. The recognition results of the original YOLOv5s model for litchi fruits are 6.03, 14.2, and 19.77 percentage points higher than those of the MobileNetv3-YOLOv4 model (82.87%), the YOLOv4-tiny model (74.7%), and the SSD with VGG model (69.13%), respectively. The recognition result of the improved model is 3.5% higher than that of the original YOLOv5s model. In conclusion, it can be concluded that the improved model has better recognition performance than the other four network models. Compared with the rest of the object detection networks on the model size, the improved YOLOv5s network is only 5.1 MB, which is the smallest. In terms of detection time, the detection frame rate of the improved model is 78.13 frames per second, which is 46.76 frames/s lower than that of the YOLOv4-tiny model but significantly higher than that of the MobileNetv3-YOLOv4 model and the SSD with VGG model. Therefore, our model has superior recognition results, model size, and inference speed as an improved lightweight object detection model.

**TABLE 6:** Detection results of different object detection algorithms on litchi images.

Models	mAP (%)	Model size (MB)	FPS
YOLOv5s_ShuffleNet_v2_ CBAM_1280_cut **(ours)**	**92.4**	**5.1**	78.13
YOLOv5s	88.9	13.7	104.17
YOLOv4-tiny	74.7	22.4	**124.89**
MobileNetv3-YOLOv4	82.87	53.7	56.83
SSD with VGG	69.13	91.1	25.66

Bold values represent the best performance exhibited among detection models.

#### The deployment of model APP

**FIGURE 10:**
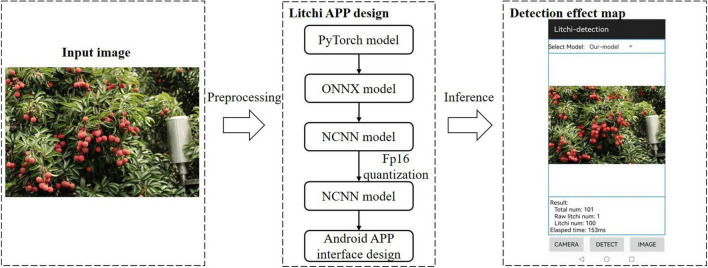
Schematic diagram of the detection scheme of the Litchi APP.

Based on the model proposed in this study, we developed a mobile application software on the Android platform that can count the number of litchi fruits at different maturity stages in the image. Figure 10 is a schematic diagram of the detection scheme of the Litchi APP. The design scheme of the software was to first convert the pt model file from the trained PyTorch model into an ONNX model. Secondly, the ONNX model was converted into the NCNN model, and then the modified model was subjected to Fp16 quantization operation to complete the final model conversion. Finally, Android Studio software’s interface development and programming were carried out. The software had the online and offline detection function for litchi images. Some results for the selected litchi images, such as the number of litchi fruits with different maturity levels, the total number of fruits in the image, and the total time spent in detecting images, will be shown in the result area after model recognition.

**FIGURE 11:**
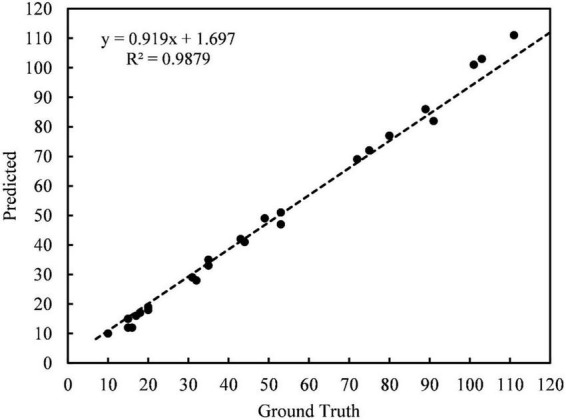
The fitted curve between the ground truth in the orchard and the predicted value of the Litchi APP.

To evaluate the detection precision by the application software, thirty images from the test set containing different maturity, light conditions, and density levels were randomly selected for detection. Each image was tested three times, and the average detection time was calculated. The test results were recorded and compared with the actual number of fruits. The fitted curve between the actual result value of the image and the software test result is shown in Figure 11. The Equation of the fitted curve is in the form of a linear function, and the correlation coefficient *R*^2^ is close to 1, indicating that the software test result value is very close to the actual result value of the image. It can be inferred that the software we developed has a high-precision detection performance in yield estimation. Meanwhile, for the detection speed of the model on the mobile phone terminal, the average time for statistical test detection on a smartphone with a Kirin990 processor is 182 ms.

## Conclusion

This study focused on litchi images collected under natural conditions. According to the growth characteristics of litchi, an object detection method and application software for estimating litchi yield in orchards were proposed and implemented. In this study, litchi images were first acquired, and the corresponding dataset was established. After that, ShuffleNetv2 was used as the backbone network of the improved model, and the CBAM module and a higher pixel model input size were introduced to improve the precision of the model. On this basis, the improved network structure was optimized to speed up the detection speed. Finally, the performance of the improved model was verified by training and comparative experiments. The main conclusions are as follows:

(1) This study used the improved model to detect litchi fruits on the test set and performed ablation experiments. The mAP of the improved model on the test set is 92.4%. Compared with the original YOLOv5s model, the mAP of the improved model presents a 3.5% improvement and 62.77% compression in model size. At the same time, the experimental results in different maturity stages, lighting conditions, occlusion methods, and density levels show better precision and robustness.
(2) Compared with other object detection models, the improved model has the highest mAP result. Regarding model size, the model specification of the improved YOLOv5s algorithm is much lower than that of other conventional algorithms, only 5.1MB. Meanwhile, the method in this paper is significantly more efficient in detecting speed than the MobileNetv3-YOLOv4 model and SSD model. Comparative experimental data show that the improved model achieved superior recognition accuracy and speed performance.
(3) A mobile application for litchi counting and yield estimation was developed based on the improved model, which realized the convenient and quick access to litchi yield information through the mobile phone. The correlation coefficient *R*^2^ between the application test and the actual results is 0.9879, which again shows the model’s accuracy in yield estimation. It can further provide technical support for the visual recognition of litchi harvesting robots in smart orchards.

## Data availability statement

The raw data supporting the conclusions of this article will be made available by the authors, without undue reservation.

## Author contributions

LW designed the experiments and wrote the manuscript. YZ and ZX collected the material data and carried out the experiments. SW analyzed the experimental results. YHL and YBL supervised and revised the manuscript. All authors contributed to the article and approved the submitted version.

## Abbreviations

YOLO: you only look once
CBAM: convolution block attention module
CAM: channel attention module
SAM: spatial attention module
AP: average precision of A class
mAP: average precision of multiple classes
FPS: frame per second
SSD: single shot multibox detector.
